# Opsin 5 mediates violet light-induced early growth response-1 expression in the mouse retina

**DOI:** 10.1038/s41598-023-44983-x

**Published:** 2023-10-19

**Authors:** Heonuk Jeong, Deokho Lee, Xiaoyan Jiang, Kazuno Negishi, Kazuo Tsubota, Toshihide Kurihara

**Affiliations:** 1 Department of Ophthalmology, Keio University School of Medicine, 35 Shinanomachi, Shinjuku-ku, Tokyo, 160-8582 Japan; 2 Laboratory of Photobiology, Keio University School of Medicine, 35 Shinanomachi, Shinjuku-ku, Tokyo, 160-8582 Japan; 3 Tsubota Laboratory, Inc., 304 Toshin Shinanomachi-ekimae Bldg., 34 Shinanomachi Shinjuku-ku, Tokyo, 160-0016 Japan

**Keywords:** Biochemistry, Molecular biology

## Abstract

Myopia is an abnormal vision condition characterized by difficulties in seeing distant objects. Myopia has become a public health issue not only in Asian countries but also in Western countries. Previously, we found that violet light (VL, 360–400 nm wavelength) exposure effectively suppressed myopia progression in experimental chick and mice models of myopia. The inhibitory effects of VL on myopia progression are reduced in retina-specific opsin 5 (*Opn5*) knockout (KO) mice. Furthermore, VL exposure upregulated early growth response-1 (*Egr-1*) expression in the chorioretinal tissues of chicks. However, the expression of EGR-1 and role of OPN5 in mice following VL exposure remain unclear. In this study, we examined whether VL exposure-induced EGR-1 upregulation depends on *Opn5* expression in the mouse retina. EGR-1 mRNA and protein expressions increased in the mouse retina and mouse retinal 661W cells following VL exposure. These increases were consistently reduced in retina specific *Opn5* conditional KO mice and *Opn5* KO 661W cells. Our results suggest that OPN5 mediates VL-induced EGR-1 upregulation in mice. These molecular targets could be considered for the prevention and treatment of myopia.

## Introduction

Myopia is caused by refractive error with axial length elongation, and it is one of the most common vision-related diseases. With increasing global prevalence, myopia has emerged as a significant public health concern both socially and economically^1–3^. Various factors, including environmental influences, dietary patterns, and genetic predisposition, have been suggested as potential contributors to the development of myopia^4,5^. Because pathological myopia can be a major cause of severe visual impairment and blindness, there is a need to find promising treatments for myopia progression^6^.

Environmental factors, particularly light, have emerged as important contributors to the progression of myopia^7–9^. Our previous research highlighted the preventive effects of violet light (VL; 360–400 nm wavelength) on myopia progression, not only in experimental animals of myopia^10–12^ but also in humans^13–16^. Furthermore, we found that VL exposure was effective in suppressing myopia progression in a transmission-dependent manner (from 40 to 100%) in a murine model of lens-induced myopia^11^. A recent study demonstrated that opsin 5 (*Opn5*)-expressing retinal cells (especially retinal ganglion cells) are needed for the refractive development pathway^12^, which suggests that targeting the OPN5 pathway could be a potential therapeutic molecular target for the future treatment of myopia.

Early growth response-1 (EGR-1), also known as ZNF268 (zinc finger protein 268)^17^, NGFI-A (nerve growth factor-induced protein A)^18^, Krox-24^19^, and ZENK^20^, is an important transcription factor involved in the development and/or injury^21–23^. EGR-1 is expressed in various cell types and activated by multiple stimuli, including growth factors, cytokines, chemokines, oxidative stress, or mitogens^24–26^. EGR-1 is involved in ocular growth and refraction^27–29^. In *Egr-1* knockout (KO) mice, longer eyes and a relative myopic shift in refraction have been reported^27^. The loss or reduction of function mutations of *ZENK* (the chicken and mouse orthologs of mammalian EGR-1) have been implicated in myopia progression. Previous studies have demonstrated that VL exposure can upregulate *Egr-1* expression in chick chorioretinal tissues^10^. However, the expression of EGR-1 and role of OPN5 under VL exposure still remain unclear. Therefore, in this study, we investigated whether the upregulation of EGR-1 induced by VL exposure was dependent on *Opn5* gene expression in mouse retinal cells, both in vitro and in vivo.

## Results

### Violet light-induced EGR-1 expression is reduced in Opn5 KO 661W cells

**Figure 1:**
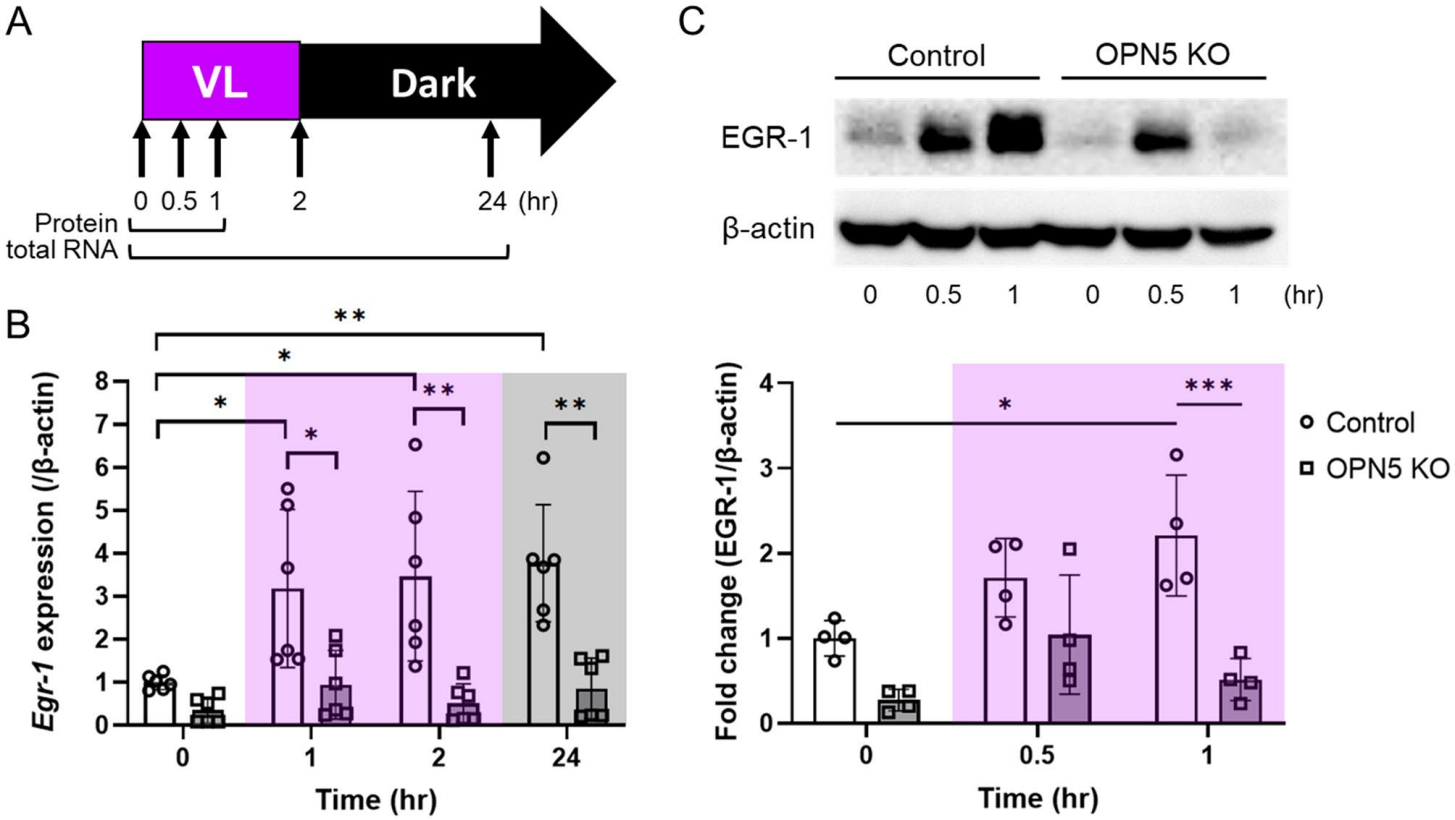
EGR-1 expression of *Opn5* KO 661w cells was not upregulated by violet light (VL) exposure. (**A**) The time points for the collection of total RNA and protein from 661w cells for western blot and real-time PCR. Cells were exposed to 280 µW/cm^2^ of VL (360–400 nm) for 2 h and subsequently cultured in the dark. At designated time points, cells were collected for western blotting or real-time PCR analyses. (**B**) *Egr-1* mRNA expression level of *Opn5* KO 661w cells before and after VL exposure (n = 6 per group). (**C**) A representative image and quantitative analysis of western blot of EGR-1 expression (n = 4 per group). The membrane images were cropped to remove irrelevant parts (Supplementary Fig. S2). Bars represent mean ± standard deviation. *p < 0.05, **p < 0.01, ***p < 0.001, two-way ANOVA with a post hoc Tukey test.

We investigated whether EGR-1 expression could be induced by VL exposure in 661W cells (Fig. 1). 661W cells and *Opn5* KO 661W cells were exposed to 280 µW/cm^2^ of VL (360–400 nm) for 2 h and subsequently cultured in the dark. At designated time points, EGR-1 mRNA and protein level in the cells were evaluated by western blotting and real-time PCR, respectively (Fig. 1A). The expression of *Egr-1* significantly increased 1 h after VL exposure (Fig. 1B). This significant increase was maintained for 24 h. However, a dramatic increase in *Egr-1* expression was not detected in *Opn5* KO 661W cells. However, this change was not observed after 24 h. Next, we checked protein expression (Supplementary Fig. S1) and a significant increase was detected 1 h after VL exposure in 661W cells (Fig. 1C). Although there was a slight increase in EGR-1 expression in *Opn5* KO 661W cells after 0.5 h of VL exposure, the dramatic induction of the expression was failed 1 h after VL exposure in *Opn5* KO 661W cells compared to that in control 661W cells.

### Violet light-induced EGR-1 expression is seen in the mouse retina

**Figure 2:**
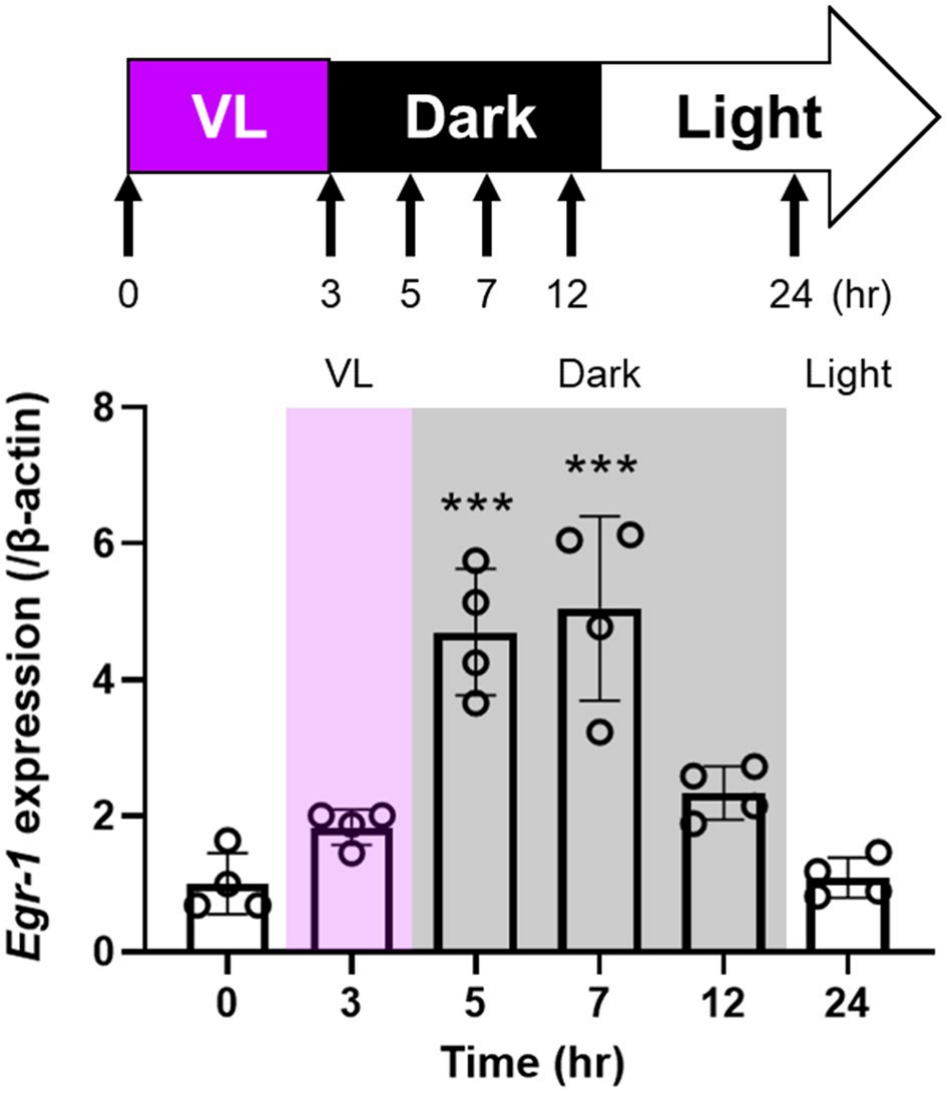
*Egr-1* expression in the wild-type mouse retina was upregulated by 5 to 7 h after violet light (VL) exposure and gradually decreased 12 h after VL exposure. Wild-type mice were housed in a 12:12-h light/dark cycle (light cycle, 00:00 to 12:00) and exposed to 400 μW/cm^2^ of VL (360–400 nm) for 3 h (9:00 to 12:00). Bars represent mean ± standard deviation (n = 4 per group). ***p < 0.001 compared to 0 h (before VL exposure), one-way ANOVA with a post hoc Tukey test.

We first determined the time point *Egr-1* expression was induced in mice after VL exposure (Fig. 2). Wild-type mice were housed in a 12:12-h light/dark cycle (light cycle, 00:00 to 12:00) and exposed to 400 μW/cm^2^ of VL (360–400 nm) for 3 h (9:00 to 12:00). Mouse retinas were collected before, and at 3, 5, 7, 12, and 24 h after VL exposure (at 9:00, 12:00, 14:00, 16:00, 21:00, and the following day at 9:00), and *Egr-1* mRNA expression was evaluated. Retinal expression of *Egr-1* dramatically increased 5–7 h after VL exposure. Its expression gradually decreased 12 h after VL exposure and reached a basal level 24 h after VL exposure. To confirm whether the increase in *Egr-1* expression is due to VL exposure or an endogenous rhythm of *Egr-1* expression, we compared *Egr-1* mRNA expression in the mouse retina housed in the dark room for 1 week with that in the retina 7 h after VL exposure. *Egr-1* expression was significantly higher in the retina exposed to VL compared to the retina in dark (Supplementary Fig. S3). We performed immunohistochemistry for EGR-1 in mouse retina before and 7 h after VL exposure (Supplementary Fig. S4). After VL exposure, EGR-1 expression was observed in the retinal ganglion cell and inner nuclear layers. Visual observation indicates an increase in EGR-1 expression in the retina after VL exposure.

**Figure 3:**
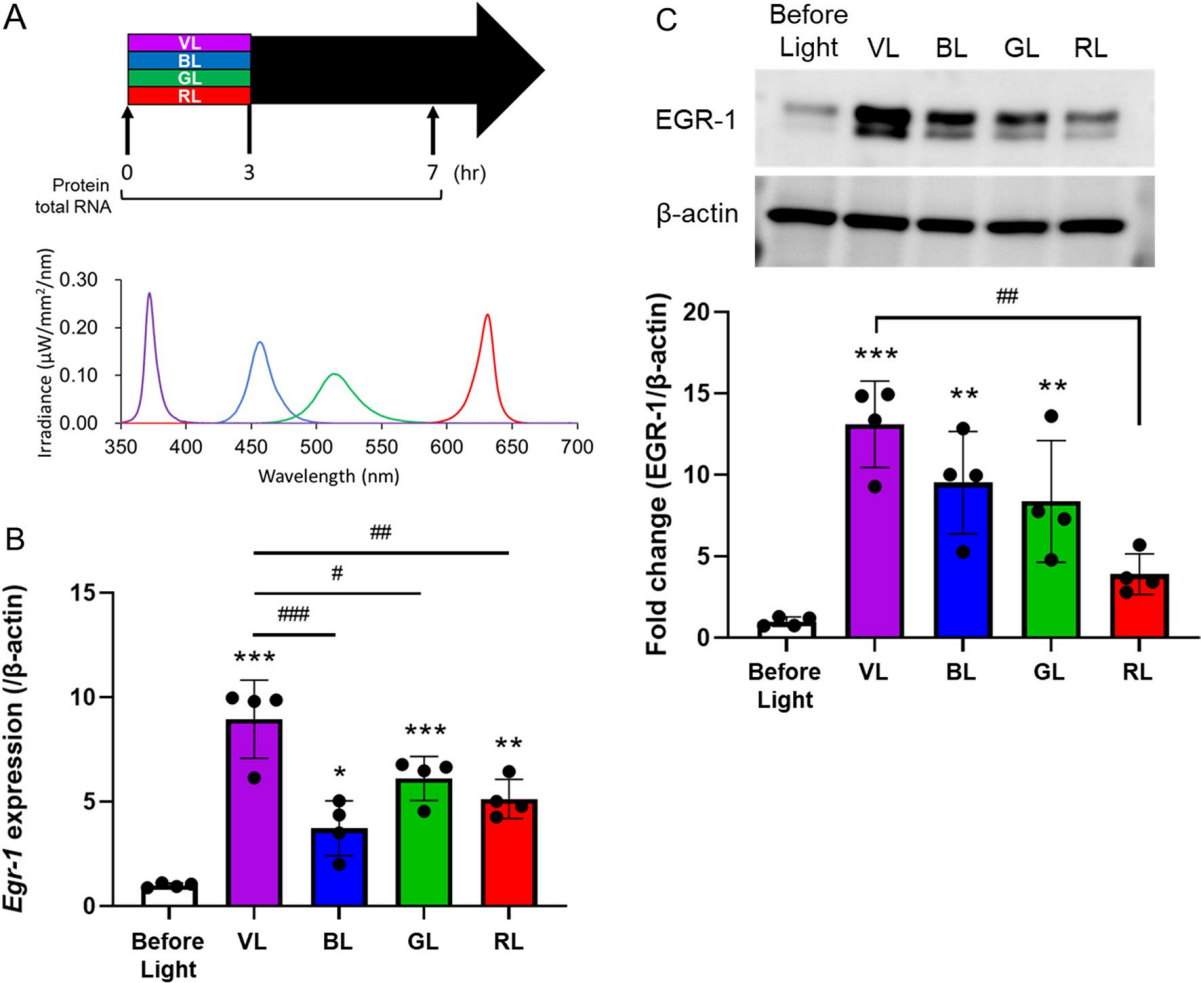
EGR-1 expression in the wild-type mouse retina was most upregulated by violet light (VL) exposure. (**A**) The irradiance of light and the time points for the collection of total RNA and protein from the retina of wild type mouse before and after 7 h light exposure for western blot and real-time PCR. Wild-type mice were housed in a 12:12-h light/dark cycle (light cycle, 00:00 to 12:00) and exposed to 400 μW/cm^2^ of VL (360–400 nm), blue light (BL, 440–480 nm), green light (GL, 500–540 nm), or red light (RL, 610–650 nm) for 3 h (9:00 to 12:00). (**B**) *Egr-1* mRNA expression level of mouse retina before and after VL exposure (n = 4 per group). (**C**) A representative image and quantitative analysis of western blot of EGR-1 expression (n = 4 per group). The membrane images were cropped to remove irrelevant parts (Supplementary Fig. S5). Bars represent mean ± standard deviation. *p < 0.05, **p < 0.01, ***p < 0.001 compared to the expression before light exposure, *p < 0.05, **p < 0.01, ***p < 0.001 compared to the expression after VL exposure, one-way ANOVA with a post hoc Tukey test.

Next, we examined whether light of various wavelengths (blue, 440–480 nm; green, 500–540 nm; red, 610–650 nm) affected EGR-1 upregulation in the retina (Fig. 3). Wild-type mice were housed in a 12:12-h light/dark cycle (light cycle, 00:00 to 12:00) and exposed to 400 μW/cm^2^ of each light for 3 h (9:00 to 12:00). Since *Egr-1* mRNA expression was higher at 7 h after VL exposure (14:00), mouse retinas were collected before, and at 7 h after light exposure (at 9:00 and 14:00), and the expression of EGR-1 mRNA and protein was evaluated (Fig. 3A). While *Egr-1* expression increased 7 h after all light exposures, VL exposure dramatically induced *Egr-1* expression in the retina (Fig. 3B). EGR-1 protein expression was significantly increased after exposure to violet, blue, and green light compared to that before light exposure (Fig. 3C). Although there was no significant difference between these lights, the shorter the exposure wavelength, the higher was the EGR-1 expression.

### Violet light-induced EGR-1 expression depends on Opn5 expression in the mouse retina

**Figure 4:**
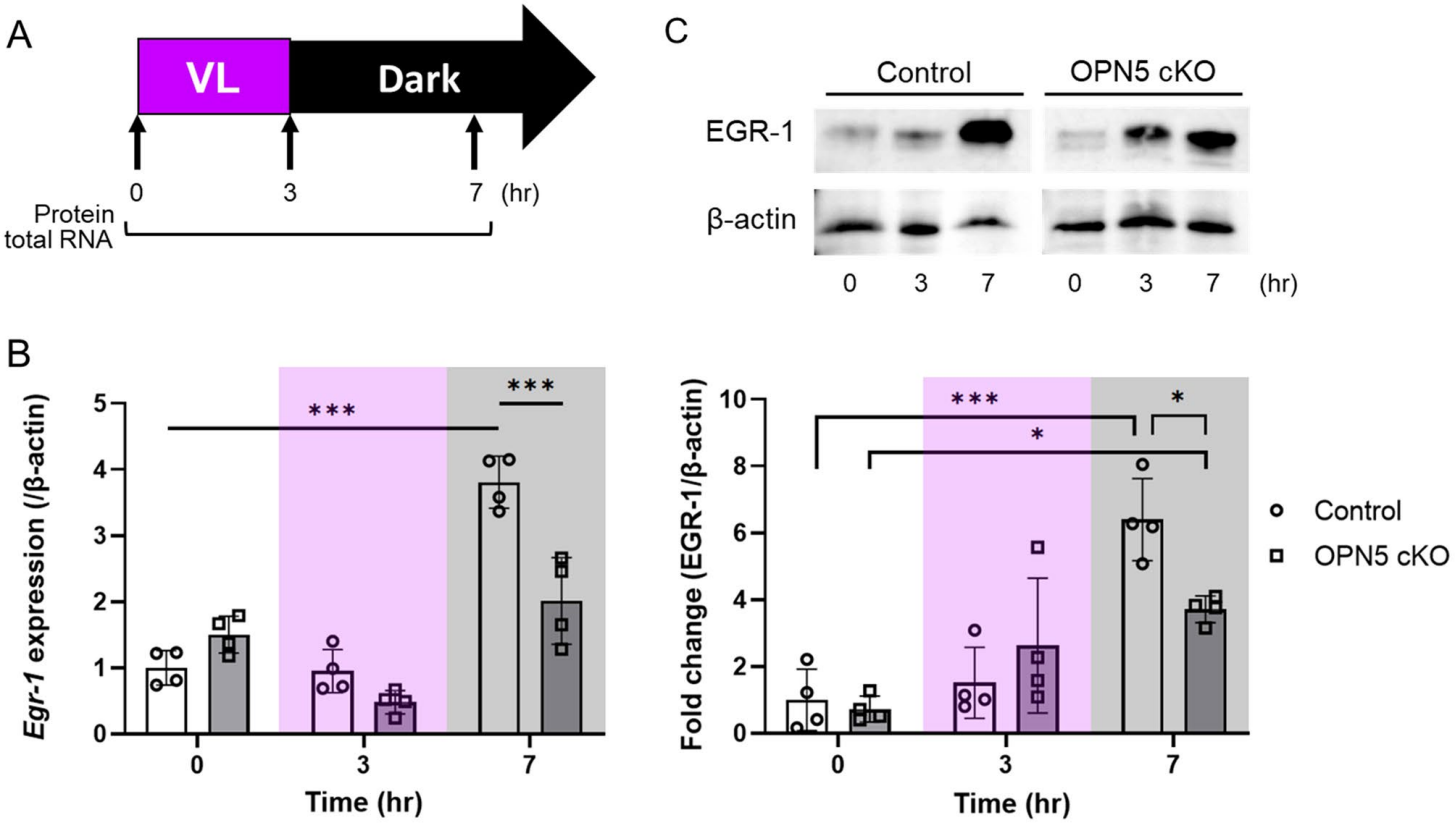
*Opn5* cKO mouse (*Opn5*^floxed/floxed^; *Chx10*-Cre^+/−^) exhibited a lower level of EGR-1 expression in the retina compared to the control mouse (*Opn5*^floxed/floxed^; *Chx10*-Cre^−/−^) after violet light (VL) exposure. (**A**) The time points for the collection of total RNA and protein from the mouse retina for western blot and real-time PCR. Mice were housed in a 12:12-h light/dark cycle (light cycle, 00:00 to 12:00) and exposed to 400 μW/cm^2^ of VL (360–400 nm) for 3 h (9:00 to 12:00). (**B**) *Egr1* mRNA expression level of *Opn5* cKO mouse retina before and after VL exposure (n = 4 per group). (**C**) A representative image and quantitative analysis of western blot of EGR-1 expression (n = 4 per group). The membrane images were cropped from different parts of the same membrane to remove irrelevant parts (Supplementary Fig. S6). Bars represent mean ± standard deviation. *p < 0.05, ***p < 0.001, two-way ANOVA with a post hoc Tukey test.

We examined whether OPN5 mediates VL-induced EGR-1 expression in the mouse retina (Fig. 4). The control and *Opn5* conditional KO mice were housed in a 12:12-h light/dark cycle (light cycle, 00:00 to 12:00) and exposed to 400 μW/cm^2^ of VL (360–400 nm) for 3 h (9:00 to 12:00). Mouse retinas were collected before, and at 3 and 7 h after VL exposure (at 9:00, 12:00, and 16:00), and the expression of EGR-1 mRNA and protein was evaluated (Fig. 4A). In terms of mRNA expression, a significant reduction in *Egr-1* expression by VL exposure was observed in retina-specific *Opn5* cKO mice after VL exposure for 7 h (Fig. 4B). This phenomenon was consistently detected in the protein expression 7 h after VL exposure (Fig. 4C). Although the expression increased in *Opn5* cKO mice after VL exposure, it was lower than that in the control group.

## Discussion

In the current study, using genetic engineering techniques, we demonstrated that VL exposure could upregulate EGR-1 expression in mouse retina and the mouse retinal 661W cells, and that VL exposure-induced EGR-1 upregulation might be highly associated with the retinal OPN5 pathway. To date, the roles of EGR-1 and OPN5 in the progression of experimental myopia have not been clearly reported. We found that these genes may be related interactively, which is of relevance to our current study.

Outdoor activities have been suggested to inhibit the progression of myopia, potentially because of the abundance of VL in outdoor light environment^8,10,30^. VL exposure has been suggested to exert anti-myopic effects in humans (from children with myopia to adults with high myopia)^13,14^. These preventive and protective effects have been observed in experimental models of myopia in mice and chicks^10,12,31^. In our previous studies, VL in the outdoor environment suppressed the development of myopia in chick and murine myopia models and in humans, and VL exposure upregulated the myopia-suppressive gene *Egr-1* both in vitro and in vivo^10^. *Egr-1* is a protein-coding gene that controls the axial elongation and progression of myopia, and *Egr-1* knockout mice exhibited longer axial length and a myopic refraction shift, suggesting a myopia-suppression-related gene^27–29^.

OPN5 (G-protein coupled receptor 136 or neuropsin) has been identified in human, mouse, and zebrafish genomes, and its expression has been detected in many neuronal tissues, including the brain and retina^32–36^. Although further studies are needed, this gene has been characterized as a UVA (380 nm)-sensitive opsin in mice. Previous studies demonstrated that *Opn5* is expressed in mouse ganglion cells. In our previous study, *Opn5*-expressing mouse retinal ganglion cells were found to be crucial for emmetropization under VL exposure^12^. In the current study, we found that this pathway regulates the expression of EGR-1, an important gene for the progression and suppression of myopia. Kato et al. demonstrated that UVA absorption by OPN5 upregulate *Egr-1* expression in chick fibroblasts^37^. Although the underlying mechanisms require further investigation, the OPN5/EGR-1 under VL exposure of the retina can be considered.

Eye growth is controlled by retinal mechanisms that function locally within the eye, causing a cascade of alterations that ultimately influence the scleral structure. Thus, the strong transcription factor, EGR-1, may be implicated as a retinal factor involved in eye growth control. EGR-1 modulates eye growth in chick retina. Specifically, glucagon-expressing amacrine cells in the chick retina respond to ocular growth via EGR-1-glucagon-dependent neurogenesis. Several pharmacologic agents prevented the downregulation of *Egr-1* mRNA expression in experimental myopia chick models such as FDM and LIM^28,38,39^. According to the accumulated evidence in mice, changes in ocular growth rates may be associated with alterations in *Egr-1* expression. As *Egr-1* KO mice have longer eyes and a relative myopia shift in refraction, regulating its gene expression may be important for myopia control^27^. Therefore, the link between VL exposure-induced EGR-1 and its possible molecular target (*Opn5*) pathway in the retina may be important for the development of promising myopia control strategies.

In our previous study, the wavelength of VL was the most effective in myopia suppression, rather than that of red, blue, or green light^12^. Consistent with this outcome, in the current study, the induction of EGR-1 expression was the highest under VL exposure. Although further studies are needed to determine why VL exposure was the most effective in suppressing myopia progression, EGR-1 expression may be one of the reasons for this outcome.

Furthermore, in our previous study, 3 weeks of oral administration of *Ginkgo biloba* extract (GBE) suppressed myopia progression in a lens-induced myopia mouse model and significantly upregulated *Egr-1* expression in the retina^40^. Additionally, GBE intake increased *Egr-1* and *endothelial nitric oxide synthase* expression in the choroid and improved choroidal blood flow, which had decreased during myopia progression. Moreover, some studies have shown that treatment with GBE increased dopamine release in the rat medial prefrontal cortex^41,42^. Dopamine, a major neurotransmitter in the retina, promotes its release and inhibits myopia development^43^. OPN5 was also found to mediate VL-dependent vascular development in the mouse eye through the regulation of the reuptake of dopamine, a neuromodulator with anti-vascular activity^44^. Chicks exposed to monochromatic short-wavelength light (370–380 nm) with lens defocusing showed less myopia and a significant increase in dopamine release^45^. The association between VL-OPN5 and dopamine may arise from a complex interaction related to the suppression of ocular growth during the progression of myopia. Another mechanism by which the VL-OPN5-EGR-1 pathway inhibits the progression of myopia may be by inducing the release of dopamine in the retina, which needs to be verified in future experiments.

*Egr-1* mRNA expression level at 24 h after VL exposure in 661W cells remained higher than the control (before VL), whereas this effect was not observed in in vivo experiment (Figs. 1B and 2). The regulation of *Egr-1* expression may involve humoral factors or circadian rhythm. Previous studies have demonstrated that EGR-1 regulates the transcription of circadian rhythm-related genes (*Bmal1*, *Cry1*, *Per1*, and *Per2*) in various cell lines and tissues^26,46–49^. Additionally, OPN5 has been identified as a mediator of local circadian rhythms in the mouse retina, cornea, and skin^50,51^. Our previous study reported that the predusk exposure to VL suppresses myopia progression via OPN5 in mice and observed the effectiveness of this suppression varies depending on the timing of VL exposure, indicating a potential influence on the circadian rhythm regulated by VL and OPN5^12^. Tao et al. reported that EGR-1 mRNA and protein levels exhibit time-dependent oscillations, decreasing during the dark cycle in mouse liver^46^. Likewise, Jackson et al. demonstrated that *Egr-1* mRNA expression in the mouse retina during normal light/dark cycles decreased in the dark cycle^52^. However, our study showed an increase in both EGR-1 mRNA and protein expression following VL exposure in the mouse retina, although this increase was followed by a subsequent decrease at 12 h (Fig. 2). Importantly, this increase was significantly attenuated in OPN5 cKO mice, suggesting a dependency of EGR-1 expression on the interaction between VL and OPN5.

In this study, we observed an increase in EGR-1 expression upon exposure to light of wavelengths other than those of VL. Light intensity can affect axial length, choroidal thickness, and refractive modulation in experimental animal models, indicating its potential involvement in the progression or suppression of myopia^53–56^. Additionally, VL exposure led to the upregulation of EGR-1 expression in *Opn5* cKO mice, although to a lesser extent than that in the control group. Previous research has reported the presence of UV-sensitive cones in the mouse retina^57,58^, and short-wavelength light exposure suppresses myopia through cone signaling^31^. These findings suggest the existence of mechanisms involving VL receptors in addition to OPN5 that contribute to the suppression of myopia. In addition, it's worth noting that OPN3 (encephalopsin) and OPN4 (melanopsin), which have peak wavelength spectral sensitivity in the blue light spectrum^36,59^, can also respond to light at wavelengths close to 400 nm. Both of these opsins are expressed in some retinal ganglion cells and may play a role in the regulation of EGR-1 expression following VL exposure. Indeed, increased EGR-1 expression was observed in both in vitro and in vivo experiments with OPN5 KO (Figs. 1 and 4). Additionally, EGR-1 expression also increased with BL, GL, and RL exposure (Fig. 3). While this study demonstrated that EGR-1 expression induced by VL is primarily dependent on OPN5, the potential influence of these photoreceptors cannot be disregarded and should be subject to further investigation.

In summary, although the therapeutic roles of EGR-1 and OPN5 have been reported in experimental myopia progression and VL research, their links have not yet been clearly determined. Based on our present results, OPN5 may mediate VL-induced EGR-1 expression in the mouse retina and mouse retinal cells. These molecular targets could be considered for the prevention and treatment of myopia.

## Materials and methods

### Cells and transfection

Murine retinal precursor cell line 661W (a kind gift from Dr. Muayyad Al-Ubaidi, University of Oklahoma Health Sciences) was cultured in culture media, Dulbecco’s modified Eagle’s medium (DMEM) low glucose (08456-65, Nacalai Tesque, Kyoto, Japan) supplemented with 10% fetal bovine serum and 1% streptomycin-penicillin at 37 °C under in a 5% CO_2_ incubator. To generate *Opn5* KO 661W cells, the cells were transfected using Lipofectamine™ 3000 transfection reagent (L3000001, Invitrogen, CA, USA) with *Opn5* or a control plasmid in culture medium without fetal bovine serum and streptomycin-penicillin. A plasmid containing the mouse *Opn5* gRNA sequence (GCTCAGGTGCATAGTCCCCC) and a puromycin resistance gene was synthesized by GenScript (NJ, USA). After 48 h, cells were cultured in culture media supplemented with 2 µg/mL puromycin (P8833, Sigma-Aldrich, MO, USA) until no infected cell death. Subsequently, cells expanded into multiple colonies. The expression of *Opn5* in transfected cells was estimated using PCR (Supplementary Fig. S7).

### Animals

All procedures were approved by the Ethics Committee on Animal Research of Keio University School of Medicine and adhered to the Association for Research in Vision and Ophthalmology Statement for the Use of Animals in Ophthalmic and Vision Research, Institutional Guidelines on Animal Experimentation at Keio University, and Animal Research: Reporting of In Vivo Experiments Guidelines for the Use of Animals in Research.

All wild-type C57BL/6J mice were obtained from CLEA Japan, Inc. The *Chx10*-Cre mouse line^60^, with the C57BL/6J genetic background, was acquired from Jackson Laboratory and crossed with the *Opn5*^floxed/floxed^ mouse line^44^ to generate *Opn5* cKO mice (*Opn5*^floxed/floxed^; *Chx10*-Cre^+/−^). Cre-negative littermates served as controls (*Opn5*^floxed/floxed^; *Chx10*-Cre^−/−^) (Supplementary Fig. S8). All mice were housed in standard transparent mouse cages, accommodating four or five mice per cage, within a temperature-controlled room set at 23 ± 3 °C, with a 12-h light–dark cycle and unrestricted access to normal food and tap water.

### Light interventions

For in vitro experiments, control and *Opn5* KO 661W cells were cultured in culture medium and maintained at 37 °C in a 5% CO_2_ incubator. Prior to VL exposure, the cells were maintained in the dark. The cells were then exposed to 280 µW/cm^2^ of VL in the range of 360–400 nm for 2 h, followed by darkness for 24 h. At designated time points, cells were collected using appropriate methods for subsequent western blotting and real-time PCR analyses.

For the in vivo experiments, mice were housed in a dedicated mouse room, where they were exposed to approximately 50 lx of white fluorescent tube light from 00:00 to 12:00 for 3 weeks prior to the light interventions. Subsequently, 6–8-week-old mice were subjected to light exposure interventions, including 400 μW/cm^2^ of VL (360–400 nm), blue light (440–480 nm), green light (500–540 nm), or red light (610–650 nm) for 3 h from 9:00 to 12:00. Spectral irradiance was measured using an OP-IRRAD-UV/VIS spectrometer (Ocean Insight, FL, USA). At designated time points, the eyes were enucleated from mice, and the retina was collected using appropriate methods for subsequent western blotting and real-time PCR analyses.

### Real-time PCR analysis

Total RNA from 661W cells and mouse retinas before or after VL exposure was isolated using TRI reagent (TR118, Molecular Research Center, Inc., OH, USA) according to the manufacturer's instructions. Reverse transcription reactions for cDNA were conducted using the ReverTra Ace qPCR RT Master Mix with a gDNA remover (TOYOBO, Osaka, Japan). Real-time PCR was performed using THUNDERBIRD SYBR qPCR Mix (TOYOBO, Osaka, Japan) on an Applied Biosystems 7500 Fast Real-Time PCR System (Applied Biosystems). The primers specific for mouse *β-actin* (housekeeping gene, forward; 5′-GGAGGAAGAGGATGCGGCA-3′, reverse; 5′-GAAGCTGTGCTATGTTGCTCTA-3′) and *Egr-1* (forward; 5′-CCACAACAACAGGGAGACCT-3′, reverse; 5′-ACTGAGTGGCGAAGGCTTTA-3′) were used. The mRNA expression levels were determined by ∆∆CT analysis.

### Western blot

Total protein from 661W cells and mouse retinas before or after VL exposure was extracted using RIPA lysis buffer (89900, Thermo Fischer Scientific, MA, USA) with protease inhibitor cocktails (1183615300, Roche Diagnostics, Basel, Switzerland). The protein concentration was measured using a BCA Protein Assay Kit (23225, Thermo Fischer Scientific, MA, USA). After heating the protein lysates with sodium dodecyl sulfate (SDS) loading buffer at 95 °C for 3 min, equal amounts of protein (20 μg) were subjected to 10% SDS-PAGE and then transferred onto PVDF membranes. The membranes were incubated with a blocking solution (PVDF Blocking Reagent for Can Get Signal, NYPBR01, TOYOBO, Osaka, Japan) and incubated with the primary antibody at 4 °C overnight. After washing with TBST buffer, the membrane was incubated with a horseradish peroxidase-conjugated secondary antibody (1:5000, GE Healthcare, NJ, USA) for 1 h at room temperature. Protein signals were detected using an ECL kit (EzWestLumi plus, ATTO, Tokyo, Japan) and visualized using ImageQuant LAS 4000 mini (GE Healthcare, IL, USA). Antibodies against EGR-1 (1:1000, 4153S, Cell Signaling Technology, Danvers, MA, USA) and β-actin (1:1000, 3700, Cell Signaling Technology) were used. EGR-1 signal was normalized for β-actin using ImageJ software (National Institutes of Health).

### Statistical analysis

The results of this study are expressed as the average ± standard deviation. Statistical analysis was performed using one- or two-way ANOVA, followed by a post-hoc Tukey’s test. Significant difference was considered when the p-value was < 0.05.

### Supplementary Information

Supplementary Figures.

## Data Availability

The datasets used during the current study are available from the corresponding author on reasonable request.
