# Supplementary material for: Opsin 5 mediates violet light-induced early growth response-1 expression in the mouse retina
Source: Sci Rep. 2023 Oct 19;13:17861. doi: 10.1038/s41598-023-44983-x (PMC10587185; doi:10.1038/s41598-023-44983-x)
Supplement: Supplementary file 1 — Supplementary Figures. [file 41598_2023_44983_MOESM1_ESM.docx]

**Supplementary information**


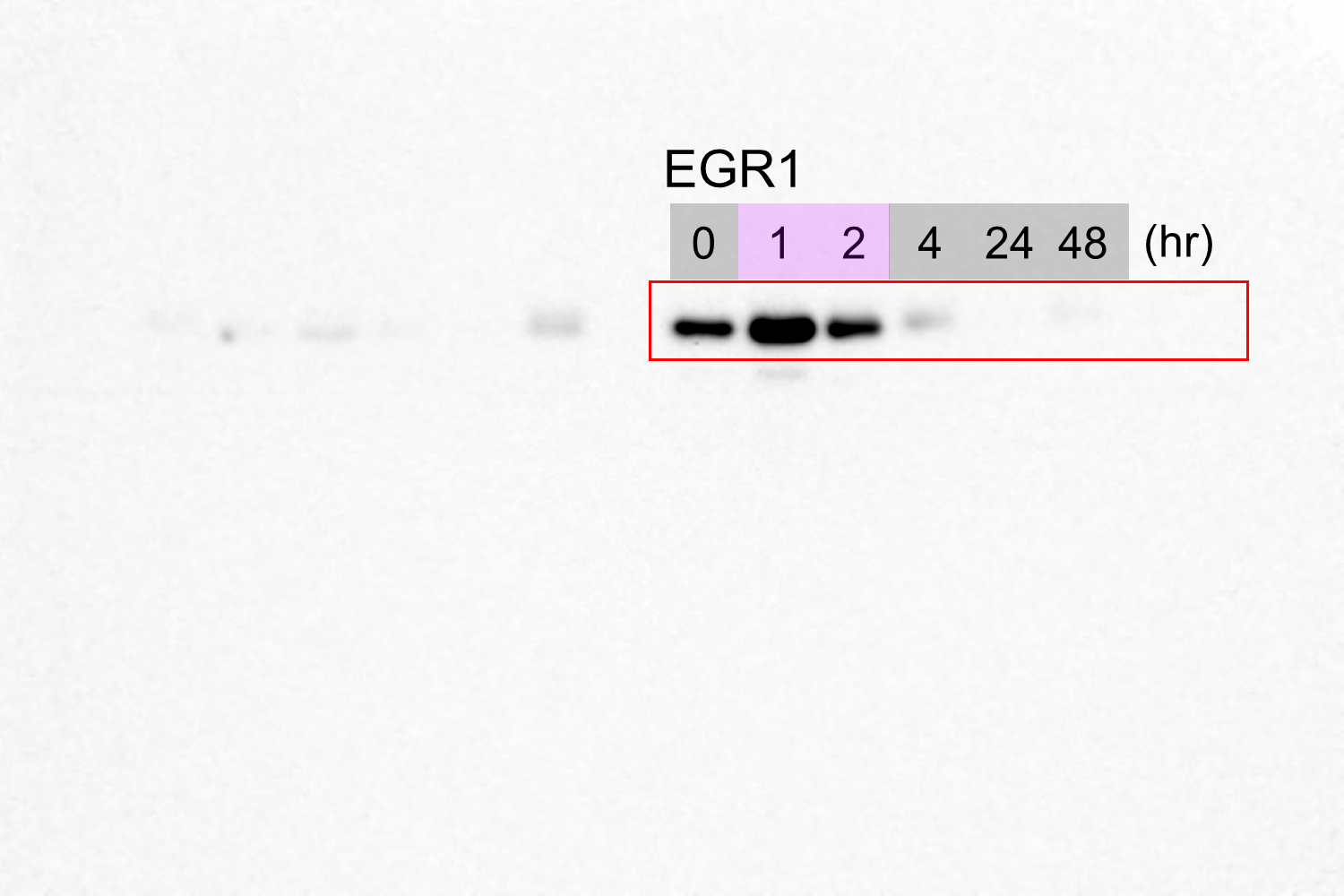

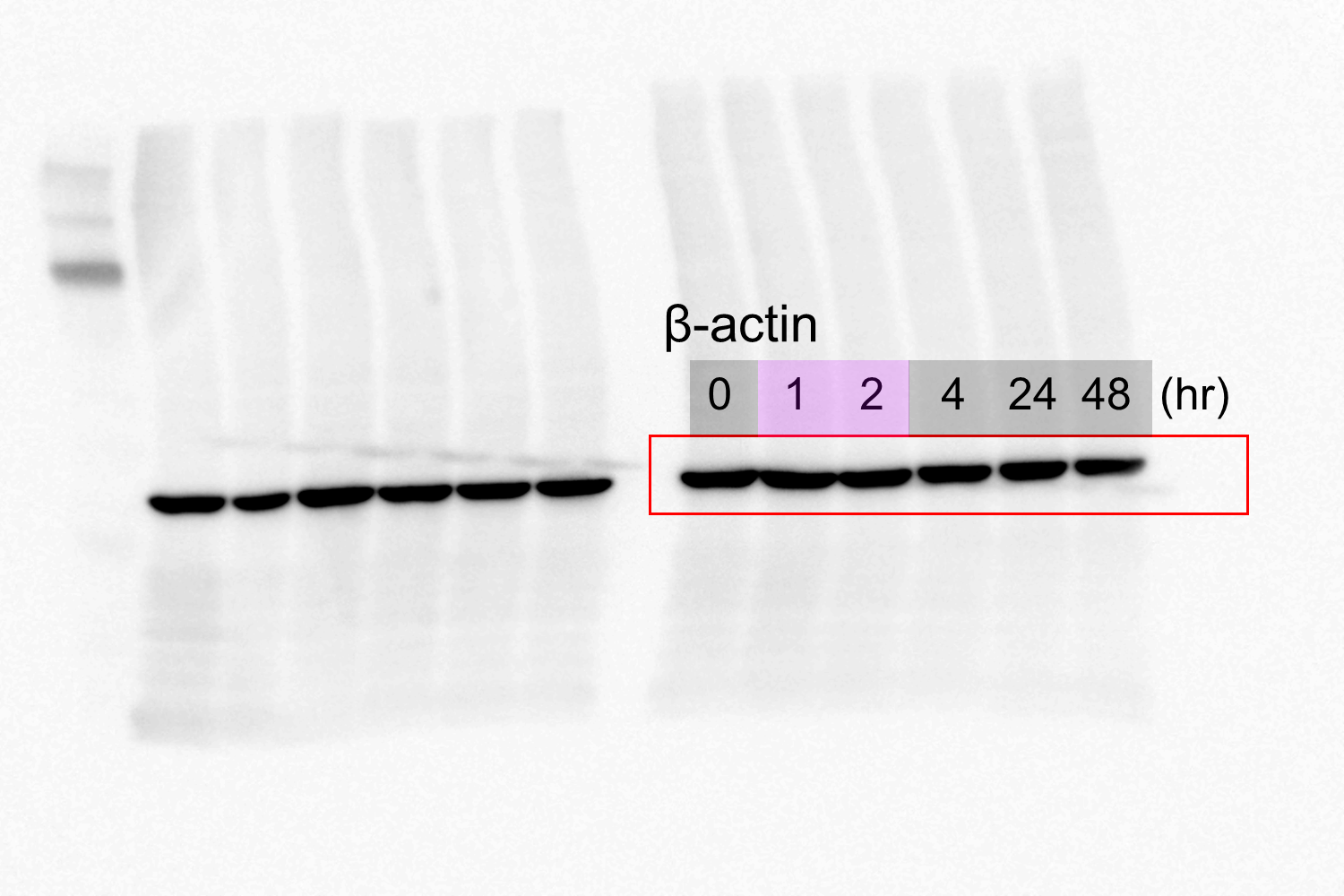


Supplementary Fig. S1. The original western blot images of EGR1 protein expression at time intervals of 0, 1, 2, 4, 24, and 48 h. 661w cells were cultured in darkness overnight and subsequently exposed to violet light exposure for 2 h, followed by incubation in darkness.


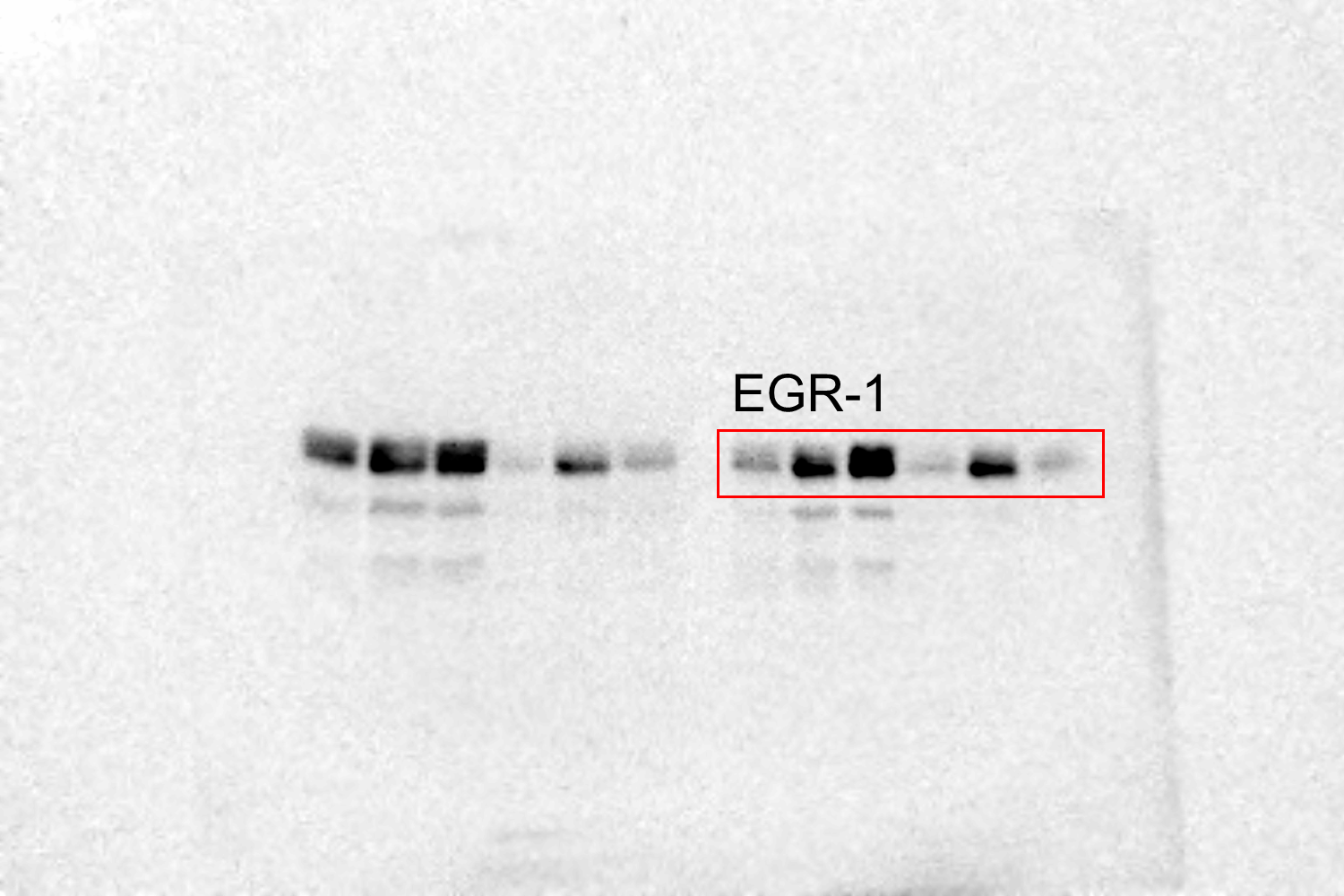

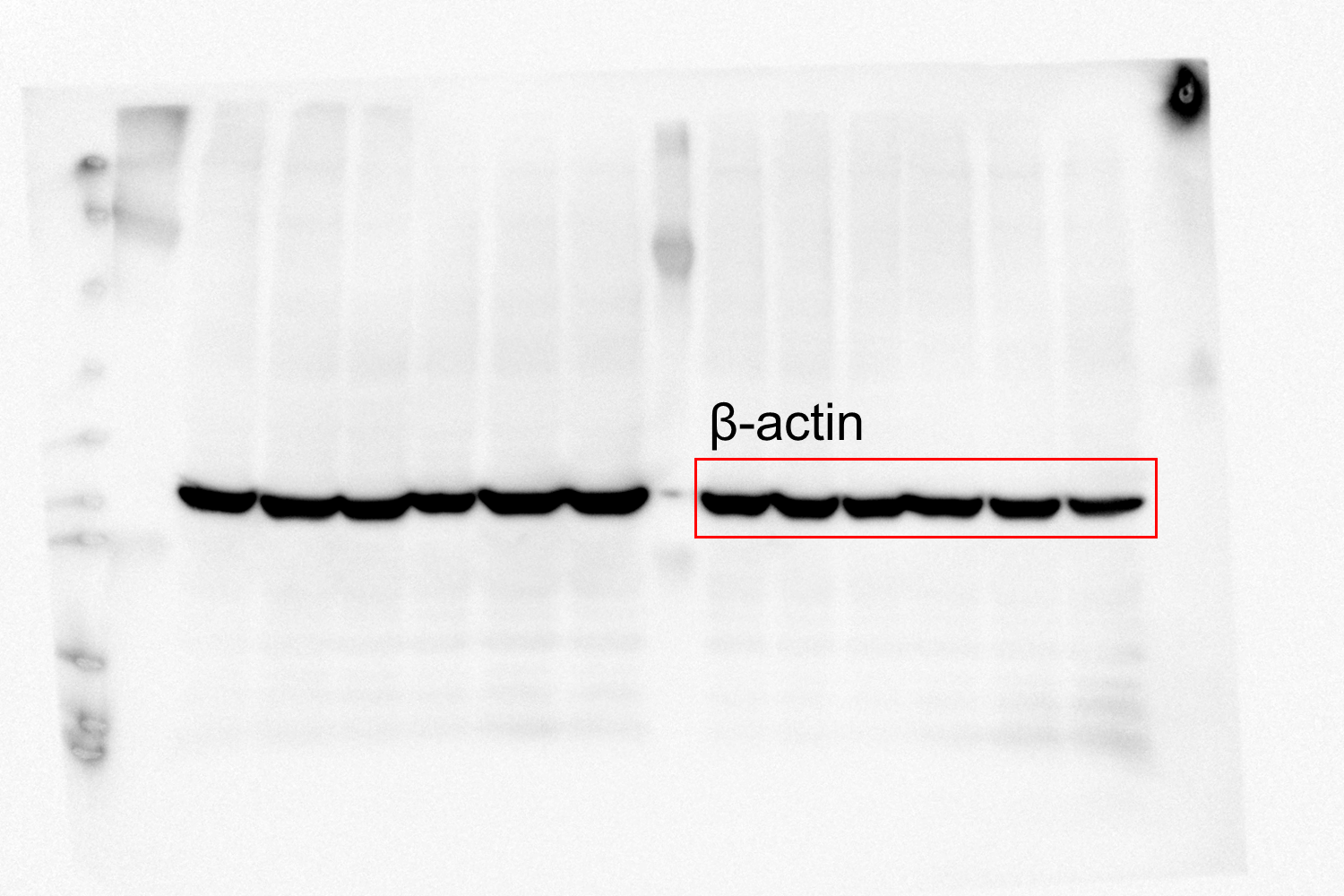


Supplementary Fig. S2. The original western blot images of Figure 1C. Red line boxes indicate the cropped membrane images.


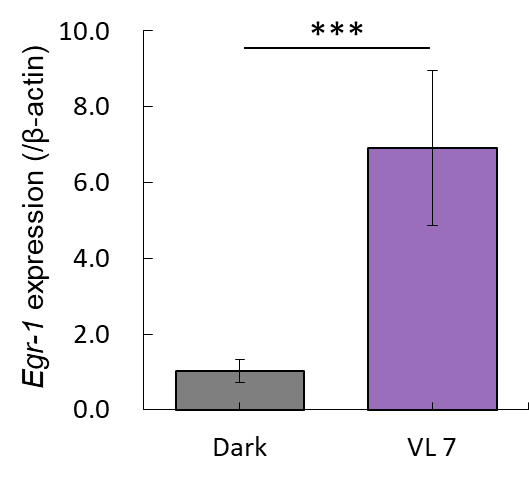


Supplementary Fig. S3. *Egr-1* expression was significantly higher in the retina exposed to VL compared to the retina in dark. Mice were housed in the dark room for one week (Dark) and *Egr-1* mRNA expression in the retina was compared to the retina 7 h after VL exposure (VL 7). n = 4 per group. Bars represent mean ± standard deviation. *** p < 0.001; Student’s two-tailed t-test.


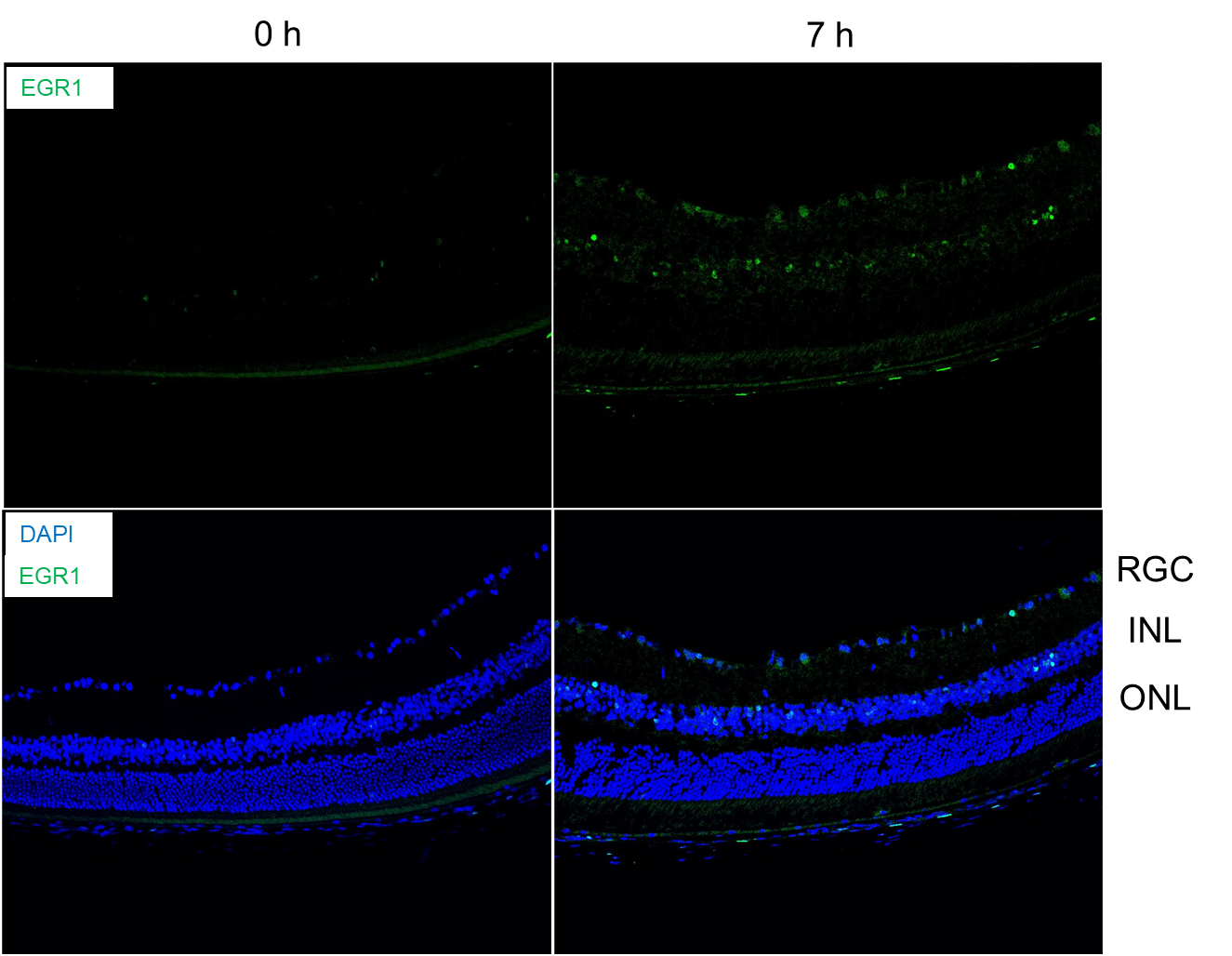


Supplementary Fig. S4. A representative immunofluorescence images of EGR-1 in mouse retina before (left panels) and 7 hours after VL exposure (right panels). Mouse eyes were enucleated before and after VL irradiation and frozen in OCT compound. Sections of eye blocks were immunostained with anti-EGR-1 antibody (1:1000, 4153S, Cell Signaling Technology, MA, USA), followed by Donkey anti-Rabbit IgG (H+L) Highly Cross-Adsorbed Secondary Antibody, Alexa Fluor 488 (1:1000, A-21206, Invitrogen, CA, USA), and DAPI (1:5000, D523, Dojindo, Kumamoto, Japan). RGC, retinal ganglion cell; INL, inner nuclear layer; ONL, outer nuclear layer


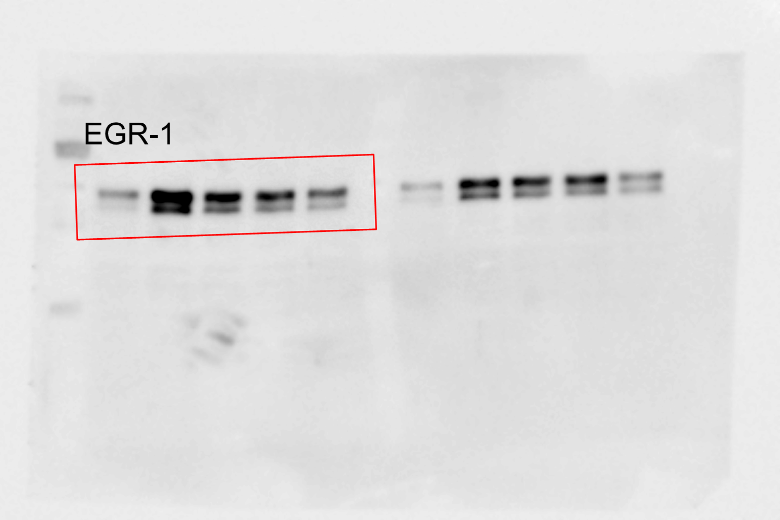

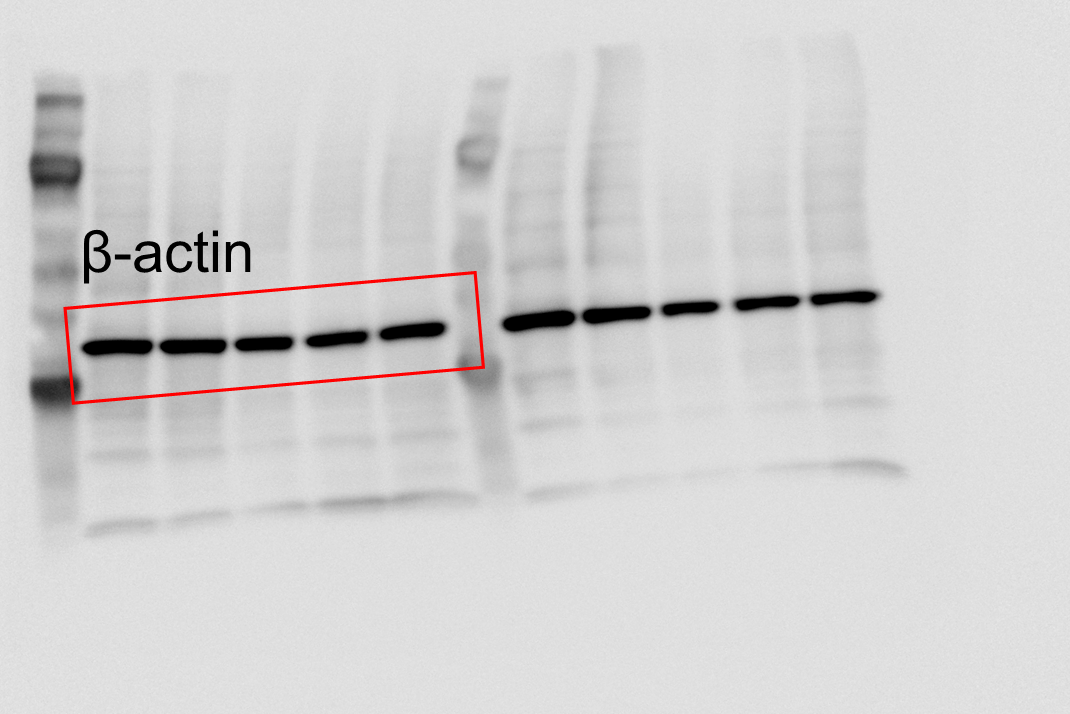


Supplementary Fig. S5. The original western blot images of Figure 3C. Red line boxes indicate the cropped membrane images.


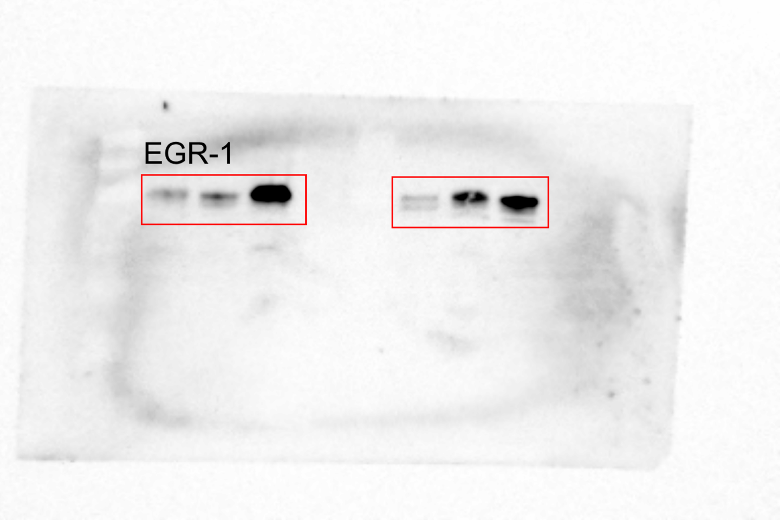

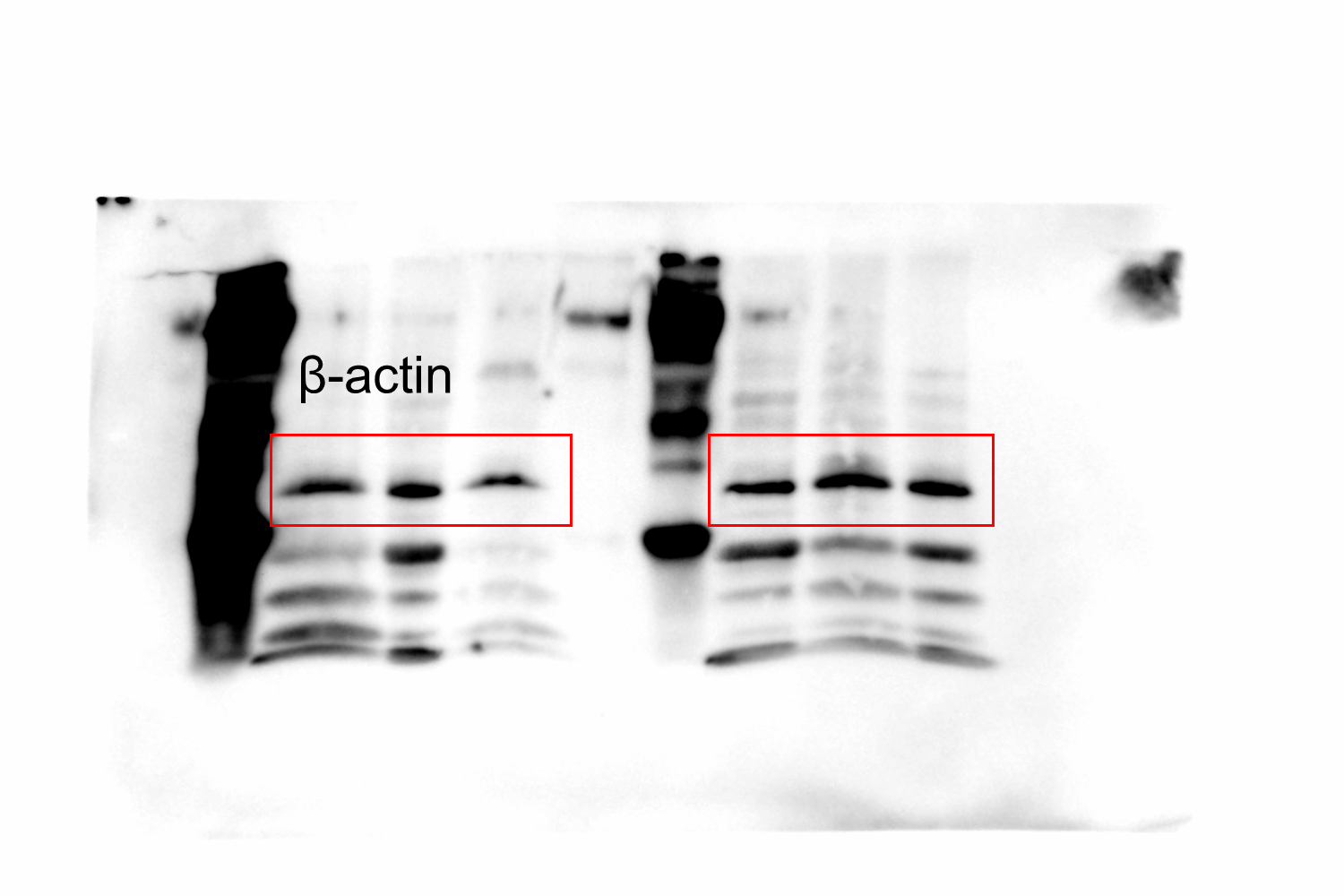


Supplementary Fig. S6. The original western blot images of Figure 4C. Red line boxes indicate the cropped membrane images.


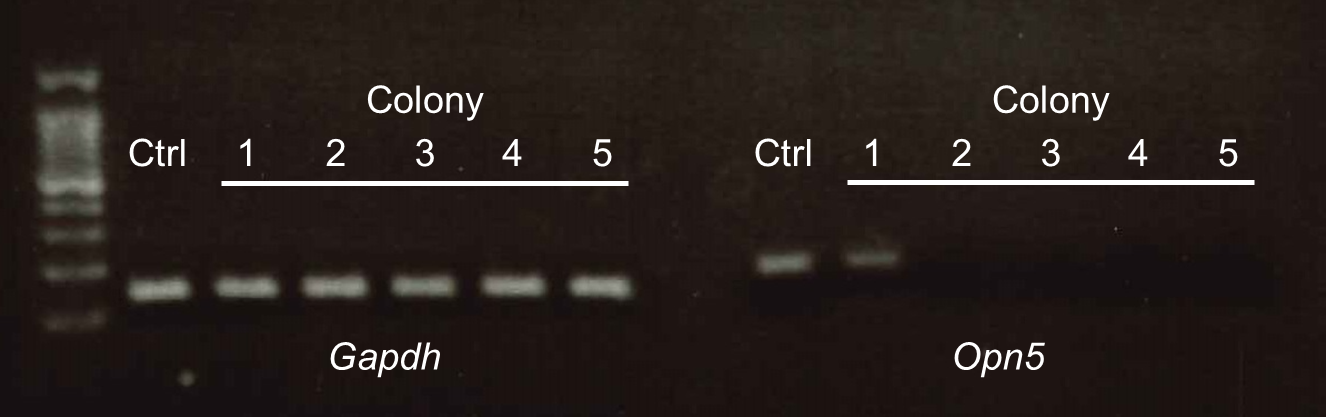


Supplementary Fig. S7. PCR result of expanded *Opn5* KO 661w cells for the assessment of transfection. Colonies 2 to 5 exhibited no *Opn5* expression. Cells from colonies 2-5 were selected for further experiments.


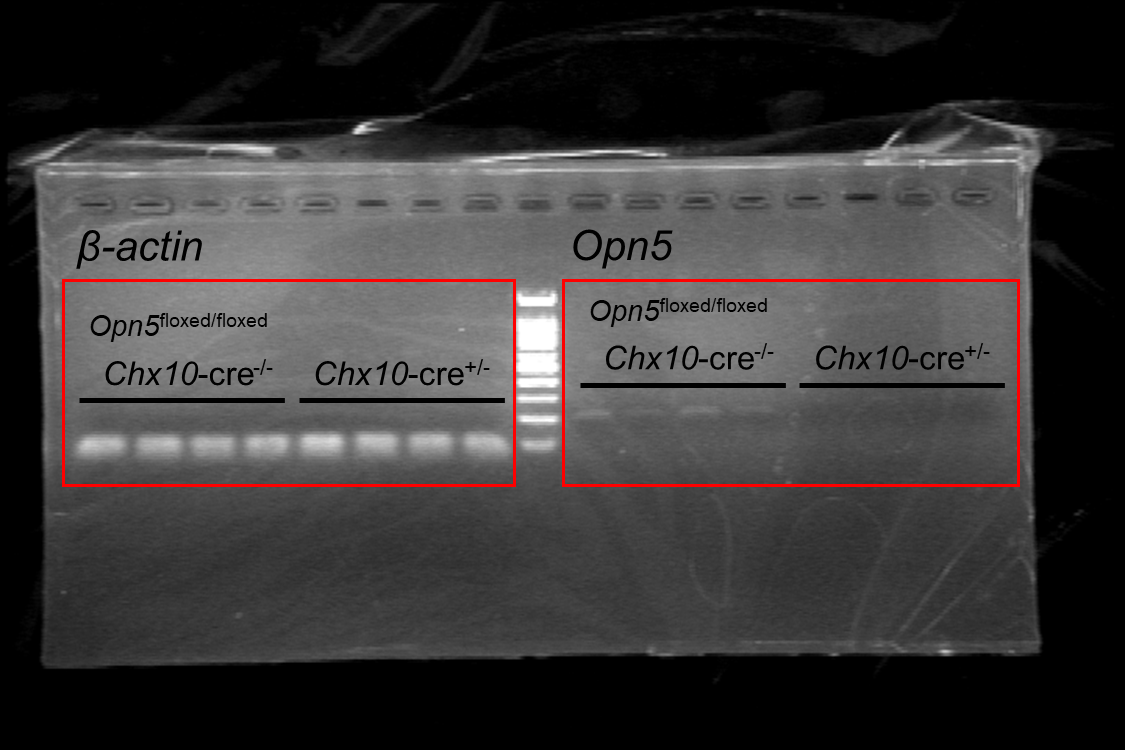


Supplementary Fig. S8. PCR results of *Opn5* mRNA expression in mouse retina. *Opn5* mRNA expression was abolished in OPN5 cKO mice. The primer sequence for *Opn5* was designed to flank exon 4 because *Loxp* site flanks exon 4 of *Opn5* (forward: TAGGCAAGCCGTTCACCATC; reverse: CTCTTCAGCCAGACCCCATAA).
